# TSPAN4-positive migrasome derived from retinal pigmented epithelium cells contributes to the development of proliferative vitreoretinopathy

**DOI:** 10.1186/s12951-022-01732-y

**Published:** 2022-12-09

**Authors:** Liangjing Wu, Shuai Yang, Hui Li, Yao Zhang, Le Feng, Conghui Zhang, Jiayi Wei, Xunyi Gu, Guotong Xu, Zhaoyang Wang, Fang Wang

**Affiliations:** 1 Department of Ophthalmology, Shanghai Tenth People’s Hospital, Tongji University School of Medicine, Shanghai, 200072 China; 2 Tongji Eye Institute, Tongji University School of Medicine, Shanghai, China; 3 Shanghai Bright Eye Hospital, Shanghai, 200050 China

**Keywords:** Migrasome, tetraspanin4, Epithelial–mesenchymal transition, Retinal pigmented epithelium, Proliferative vitreoretinopathy

## Abstract

**Background:**

Proliferative vitreoretinopathy (PVR) is a blind-causing disease initiated by the activation of retinal pigmented epithelium (RPE) primarily induced by TGF-β families. Migrasome is a recently discovered type of extracellular vesicle related to cell migration.

**Results:**

Here, we used ex vivo, in vitro, and in vivo models, to investigate the characteristics and functions of migrasomes in RPE activation and PVR development. Results indicated that the migrasome marker tetraspanin-4 (TSPAN4) was abundantly expressed in human PVR-associated clinical samples. The ex vivo model PVR microenvironment is simulated by incubating brown Norway rat RPE eyecups with TGF-β1. Electron microscope images showed the formation of migrasome-like vesicles during the activation of RPE. Further studies indicated TGF-β1 increased the expression of TSPAN4 which results in migrasome production. Migrasomes can be internalized by RPE and increase the migration and proliferation ability of RPE. Moreover, TSPAN4-inhibited RPE cells are with reduced ability of initiating experimental PVR. Mechanically, TSPAN4 expression and migrasome production are induced through TGF-β1/Smad2/3 signaling pathway.

**Conclusion:**

In conclusion, migrasomes can be produced by RPE under PVR microenvironment. Migrasomes play a pivotal role in RPE activation and PVR progression. Thus, targeting TSPAN4 or blocking migrasome formation might be a new therapeutic method against PVR.

**Supplementary Information:**

The online version contains supplementary material available at 10.1186/s12951-022-01732-y.

## Background

Proliferative vitreoretinopathy (PVR) is the main complication of rhegmatogenous retinal detachment (RRD) which results in vision loss [1–3]. During the current COVID-19 epidemic, studies in various countries have shown that the incidence of severe PVR in RRD patients has significantly increased (from about 4.5–6.9% to about 9.5–31.6%) due to the delayed treatment. The postoperative visual outcomes of these patients were also significantly worse than before [4–7].

The treatment of PVR remains a clinical challenge. Drug therapy is mostly limited to prevention rather than treatment. The efficacy is uncertain, and there are potential complications (e.g., the use of corticosteroids to prevent PVR can lead to high intraocular pressure, cataract progression, etc.) [8]. Surgery is currently the main method for the treatment of PVR, but the surgical treatment is traumatic (i.e. retinotomy/retinectomy may be required) and the surgical procedure is too complicated and requires well-experienced doctors and advanced surgical equipment. The prognosis is still poor even if the retina is reattached by surgery [9]. Therefore, in this special historical period, it is particularly important to further clarify the pathogenesis of PVR.

After RRD, retinal pigmented epithelium (RPE), a monolayer of cells physiologically located between the neural retina and Bruch’s membrane, was exposed to an abundant of variety of cytokines, including VEGF, TGF-β, and PDGF-BB as a result of breakdown of blood-retina-barrier and the formation of retinal tear [10–14]. The RPE cells were then activated and underwent progress named epithelial-mesenchymal transition (EMT). After EMT, RPE transdifferentiates into myofibroblast cells which finally form contractile subretinal or epiretinal membranes that contract and lead to traction retinal detachment [13, 15]. Of these cytokines, TGF-β, a potent EMT inducer, is one of the most up-regulated and abundant cytokines which powerfully activates RPE and is the most widely-used cytokine by researchers to simulate the ex vivo and in vitro PVR microenvironments.

In PVR microenvironment, the originally well-organized junction complex of RPE cells is gradually compromised and disrupted [16–19]. Though traditional intercellular communication approach was disrupted with the breakdown of cell-cell junction, extracellular vesicles (EVs), an alternative intercellular communication method [20, 21], might be capable of transporting messages between RPE. There are several types of EVs, which are categorized based on their mechanism and size [22]. Ectosomes (such as microvesicles and oncosomes) are formed via the direct budding of the plasma membrane, with a diameter range of 100–1000 nm. Exosomes are a smaller subset of EVs (30–150 nm) formed by the endocytic pathway. Recently, Yu et al. discovered a special subset of budding vesicles called migrasomes, which are formed along the retraction fibers (RFs) of migrating cells and are 0.5–3 μm in diameter [23, 24]. Subsequent work discovered that migrasomes can serve as a platform for cell–cell communication and initiate an enhanced migration ability in recipient cells under physiological conditions [25–27]. Migrasomes play an indispensable role in cell migration that guides a cluster of cells named dorsal forerunner cells (DFCs) to the right position in Kupffer’s vesicle [27]. Specifically, knockout of TSPAN4, a migrasome marker, in zebrafish led to DFCs misallocation that formed impaired Kupffer’s vesicle morphogenesis, while injection of exogenous migrasomes restored the asymmetrical phenotype. Besides, migrasome also plays an important role in maintaining mitochondrial quality by intaking and disposing of damaged mitochondrial in migrating cells suffered from excessive mitochondrial stress [28]. Moreover, migrasome are proven to transfer signal molecules, such as mRNAs. Scientists discovered that migrasomes transported mRNAs into recipient cells and then translated into proteins which can functionally modify the recipient cells [26]. There are still many puzzles in migrasome function to investigate [29]. Considering the importance of RPE migration in PVR progression, here we aimed to uncover whether migrasomes also have efforts in RPE migration and activation, which finally result in PVR development.

## Results

### Identification of migrasome and related marker expression in PVR microenvironment and normal retina

**Fig. 1:**
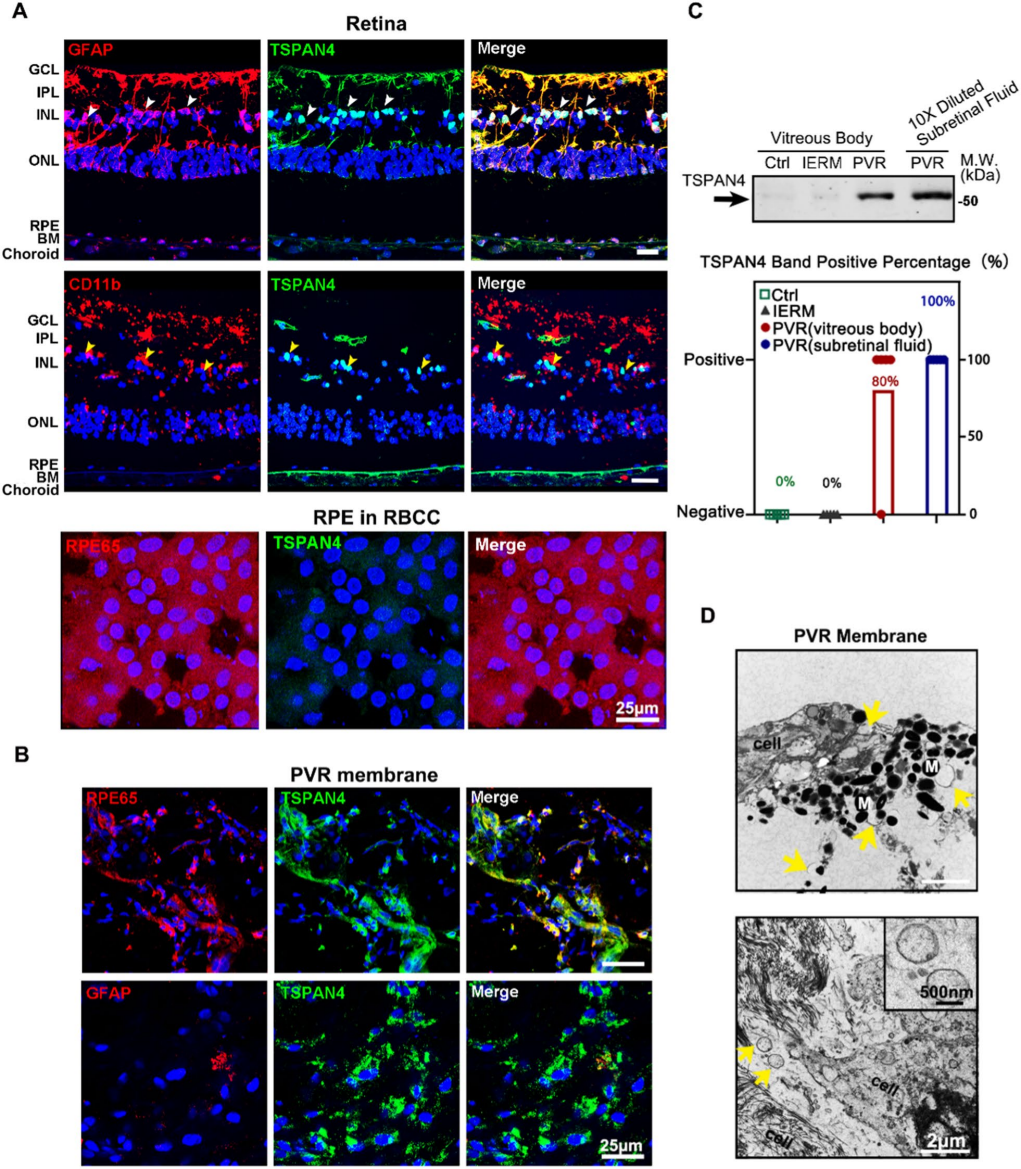
Expression of TSPAN4 in human PVR membranes clinical sample, normal donated retina and vitreous bodies. **A** Immunofluorescence analysis of TSPAN4 (green) in normal donated retina section and normal RPE in RBCC. RPE65 is the marker of the RPE cells. The Müller cells are labeled with GFAP (red) and microglia cells labeled with CD11b (red). Scale bars, 25 μm. **B** Immunofluorescence analysis of TSPAN4 (green) in PVR membranes. Scale bars, 25 μm. **C** The expression of TSPAN4 in vitreous bodies from donated human eyes (normal control group), idiopathic macular epiretinal membrane (IERM) and PVR patients, and subretinal fluid from PVR patients were detected by western blots. The percentage of TSPAN4^+^ samples, as detected by western blot, in each group was calculated. n = 5 in each group. **D** Observation of PVR membrane by TEM. Yellow arrows indicated the vesicles out of the cell. The extracellular vesicles released by cells are shown in the amplified picture. GCL, ganglion cell layer; IPL, inner plexiform layer; INL, inner nuclear layer; ONL, outer nuclear layer; M, melanin

To examine whether migrasomes exist in retina, we first explored the expression of TSPAN4, the marker of migrasome, in normal retina. Retina is a beautifully layered structure tissue composed of a dozen different types of cells. After double-stained with markers of müller cell (GFAP), microglia (CD11b), and RPE (RPE65), it is clearly shown that TSPAN4 is mainly expressed in müller cells, rather than in microglia or RPE in human donated RPE–Bruch’s membrane–choriocapillaris complex (RBCC) (Fig. 1A).

Besides, to uncover whether TSPAN4 is up-regulated in the PVR microenvironment, the TSPAN4 expression was examined on PVR membranes obtained from surgery. The RPE cells were distinguished by RPE65, the marker of RPE. Results showed compared with the normal donor eyes, the RPE in the PVR membrane highly expressed TSPAN4 (Fig. 1B). The limited co-localization of TSPAN4 and GFAP excluded the existence of TSPAN4^+^ müller cells in PVR membrane. What’s more, to further determine whether RPE releases migrasomes, we tested whether TSPAN4 is found in the vitreous body and subretinal fluid of PVR patients (Fig. 1C). Statistically, the percentage of TSPAN4^+^ sample, as detected by western blot, in each group is illustrated (Fig. 1C). The results of western blot revealed the expression of a specific band (~ 50 Kd) in 4 of the 5 PVR vitreous body samples and all 5 subretinal fluid samples compared to 0/5 in the donor eyes and idiopathic macular epiretinal membrane (IERM) samples (Fig. 1C). In addition, the subretinal fluid with 10 ×dilution express a more abundance of TSPAN4 than the vitreous body. This result indicated that the over-expression of TSPAN4 is in a secretary manner in PVR microenvironment. We also observed the ultrastructure of the PVR membrane by transmission electron microscope (TEM) (Fig. 1D). Outside of the plasma membrane, vesicles with a diameter of around 1000 nm were also observed (yellow arrows). These results demonstrate that 1000 ~ nm EVs exist in PVR membranes. According to previous researches, the diameter of migrasome was usually ~ 1 μm and was slightly different in size across different cell types [23, 27]. (i.e. the diameter of migrasome derived from mouse bone marrow-derived macrophage is around 800 nm, while in mesendodermal cell-derived migrasome is around 1000 nm.) Thus, the EVs in Fig. 1D discovered in PVR membrane met the size of migrasome. Besides, western blots and immunofluorescence have proved that migrasome markers were expressed in these EVs under PVR microenvironment (Fig. 1B, C). TEM identified externally deposited EVs to be reminiscent of migrasomes. Taken together, we believe the vesicles marked by yellow arrows are migrasomes.

### TGF-β1 treatment induces migrasome formation and TSPAN4 expression in rat RBCCs

**Fig. 2:**
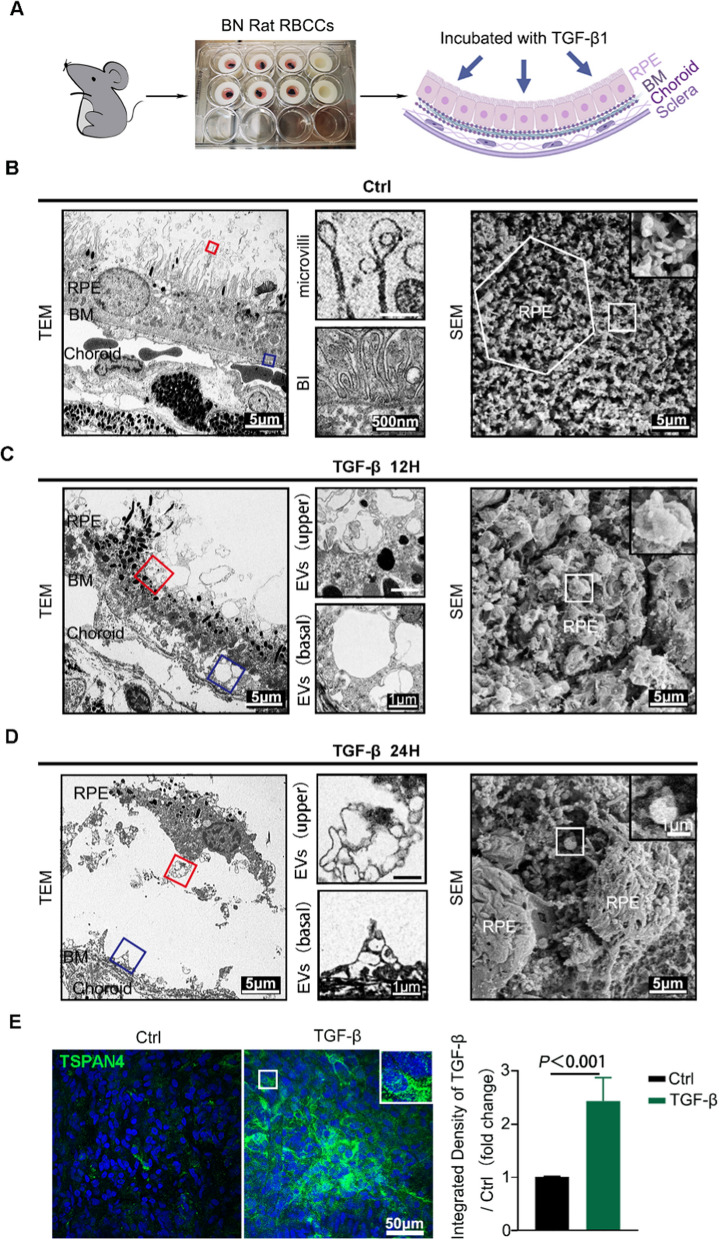
Observation of migrasome formation and production from TGF-β1-treated retinal pigmented epithelium (RPE) cells. **A** A flow chart of ex vivo model preparation with rat RBCCs. **B** TEM and SEM of RPE in the normal control group. TEM shows the orderly structure of apical microvilli, basal infoldings (BI), and extracellular vesicles (EVs) are visualized at higher magnification on the right portion of the figure. SEM visualizes the apical side of RPE in normal control group. The white hexagonal outline is a single cell. The structure of extracellular vesicles is visualized at higher magnification in the boxed areas. **C** TEM and SEM of RPE in TGF-β1 treatment for 12 h. TEM shows RPE transformation and vesicles forming from microvilli and BI. SEM visualizes EVs formation at the apical side of RPE. The structure of extracellular vesicles is visualized at higher magnification in the boxed areas. **D** TEM and SEM of RPE in TGF-β1 treatment for 24 h. TEM shows the migrated RPE away from Bruch’s membrane (BM). SEM visualizes the transformation of RPE and releasing EVs at the apical side of RPE. The structure of extracellular vesicles is visualized at higher magnification in the boxed areas. **E** Confocal images of control and TGF-β1-treated RBCCs labeled with TSPAN4 (green). The integrated density of figures was measured by Image J and shown in chart. Scale bar, 50 μm. RBCCs, retinal pigment epithelium–Bruch’s membrane–choriocapillaris complex. TEM, transmission electron micrograph; SEM, scanning electron micrograph; BM, Bruch’s membrane; BI, basal infoldings

To visualize the time-dependent morphology change of RPE and production of RPE-derived migrasomes in situ, we observed the RBCC of brown Norway rats incubated with TGF-β1 for different time intervals. The incubation process is illustrated in Fig. 2A. The spatiotemporal ultrastructure changes of RPE under PVR microenvironment and migrasome formation were observed via TEM and scanning electron microscope (SEM). The normal RPE cells were aligned in good order and presented a high capacity of phagocytosis, including engulfing and degrading the outer segments of photoreceptors (Fig. 2B). Vesicles with a diameter of more than 500 nm were observed. Incubation of RBCCs with TGF-β1 led to disordered cytoplasmic structures and disorganized endocytosis structures, which were mainly exhibited as transformed microvilli and basal infoldings. Physiologically, RPE is a mono-layer of well-polarized cells with elaborate microvilli and basal infoldings that are supported by actin filaments. Upon EMT, actin polymerization and protrusion activity occur. After being stimulated with TGF-β1 for 12 h, enlarged and extended vesicles started to appear on the tips or at the intersections of tangled microvilli. The disorganized basal infoldings, surrounded by plenty of vesicles (500 nm–2 μm), were observed at the bottom of the RPE cells (Fig. 2C). After 24 h, RPE cells start to lose contact with the Bruch’s membrane, whereas the filopodial protrusions decorated with EVs remained at the Bruch’s membrane (Fig. 2D). These EVs met the size of migrasome and are similar to the morphology of migrasome as described recently [23]. Therefore, these EVs are deemed as migrasome. We also examined the expression of migrasome marker TSPAN4 in RBCCs via immunofluorescence and found that the TGF-β1-treated group displayed higher labeling (*p < *0.001) for TSPAN4 compared to control group (Fig. 2E).

### Migrasomes produced by RPE cells are accompanied by the migratory process of RPE

**Fig. 3:**
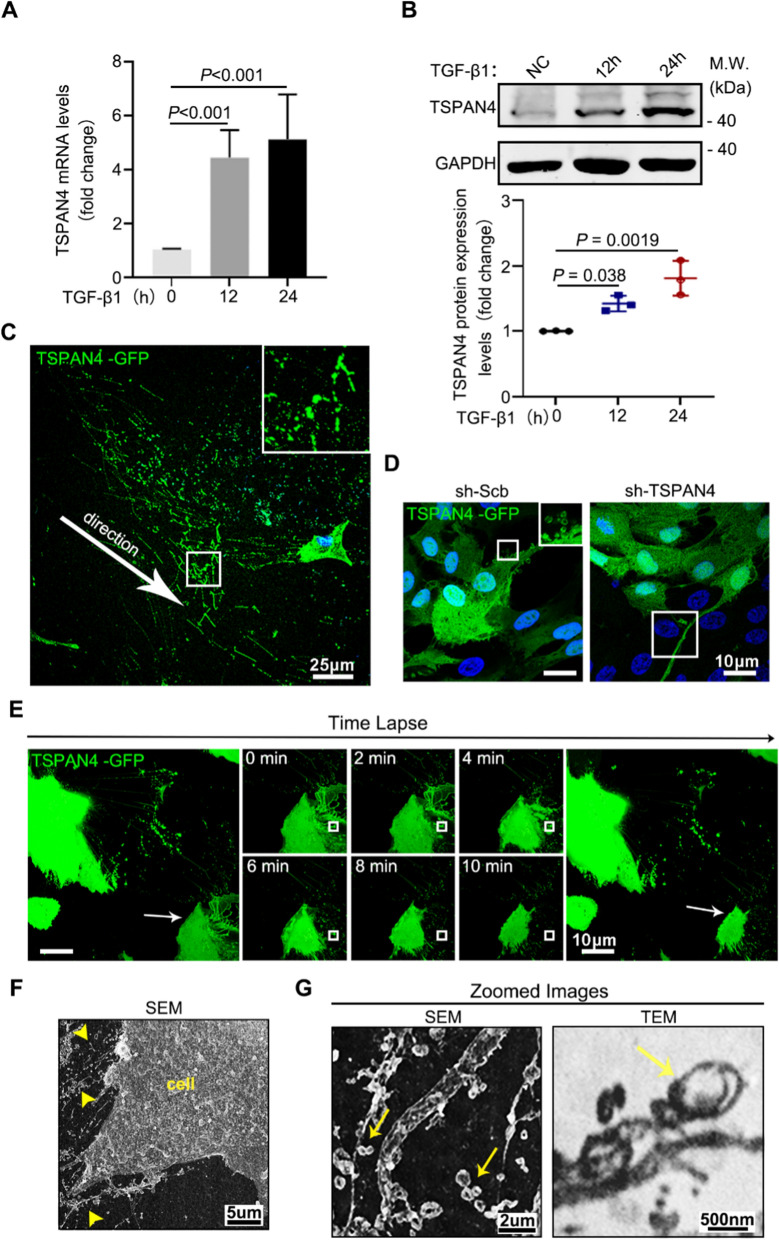
Pattern of migrasome in ***vitro*** cultured RPE cells. **A, B** RPE cells were treated with TGF-β1. The expression of TSPAN4 was then analyzed by western blots and quantitative reverse transcription polymerase chain reaction (RT-qPCR). Qualified protein expressions are shown. **C** RPE cells were infected with TSPAN4-GFP lentivirus. Migrasomes and retract fibre (RF) were observed by confocal microscope. Scale bars, 25 μm. **D** TSPAN4-GFP RPE cells were transfected with scramble shRNA (sh-Scb) or TSPAN4-inhibiting shRNA (sh-TSPAN4). Cells were then visualized by confocal microscope. Scale bars, 10 μm. **E** Time-lapse photography of TSPAN4-GFP transfected RPE cell. Retract fiber extension, migrasome formation and degradation are shown chronologically. **F** The TSPAN4-GFP transfected RPE cells were seeded on a Transwell insert. After cultured for 48 h, the Transwell membrane was harvested and subjected to SEM. Migrasomes and retract fibers were marked by yellow arrowhead. **G** Magnified images were captured by SEM and TEM. Migrasomes were marked by yellow arrows

It was found that a pronounced up-regulation of TSPAN4 upon TGF-β1 stimulation in RPE cells cultured in vitro (Fig. 3A, B). To further confirm the vesicles released by TGF-β1-treated RPE in rat RBCC are migrasomes, we further compared it with the migrasome induced in vitro by overexpressing TSPAN4. In vitro cultured RPE cells transfected with Lentivirus-TSPAN4-GFP (Lv-TSPAN4-GFP) showed a similar EVs and RFs pattern with the rat RBCC model: the long green RFs and vesicles that were left behind the migrated RPE, suggesting migrasomes were produced by these migrating RPE (Fig. 3C). When we zoomed in and put emphasis on the released stretches, over 100 nm-sized vesicles can be visualized at the bifurcation of the RFs in the amplified figure (Fig. 3C, D), which is consistent with a previous study [23]. Integrin-α5, NDST1 and EOGT are verified to be specific marker of migrasome [30]. We tested those markers in the supernatant of Lv-TSPAN4 and Lv-control group and proved migrasome existence (Additional file 1: Fig. S1A). Therefore, we defined these vesicles as migrasome. Moreover, knockdown of TSPAN4 by shRNA decreased the expression of migrasome-specific markers (Additional file 1: Fig. S1B) and immunofluorescence showed the disruption of migrasomes biogenesis in sh-TSPAN4 group (Fig. 3D), indicating an indispensable role of TSPAN4 in migrasome biogenesis. Besides, time-lapse photography was applied to dynamically observe the migrasome during RPE migration (Fig. 3E). RFs extension, migrasome formation and degeneration were gradually observed in RPE cells transfected with GFP labeled TSPAN4. These results strongly suggested the biogenesis of migrasome is accompanied by the migratory process of RPE.

TEM and SEM were further used to visualize the structure of RPE-derived migrasomes in detail. RPE cells were cultured in Transwell plates. The Transwell membrane was then harvested and subjected to SEM observation. The SEM result showed that plenty of migrasomes were aggregated at the plasma membrane, with some left at the tip of the extended retraction fiber (yellow triangular arrow) (Fig. 3F). When zoomed in, both SEM and TEM results showed that migrasomes (yellow arrowhead) were attached to the RFs (Fig. 3G). Compared with exosome (less than 100 nm), the diameter of the migrasomes measured up to 1 μm.

### TSPAN4-induced migrasome can be phagocytosis by RPE and activates RPE

**Fig. 4:**
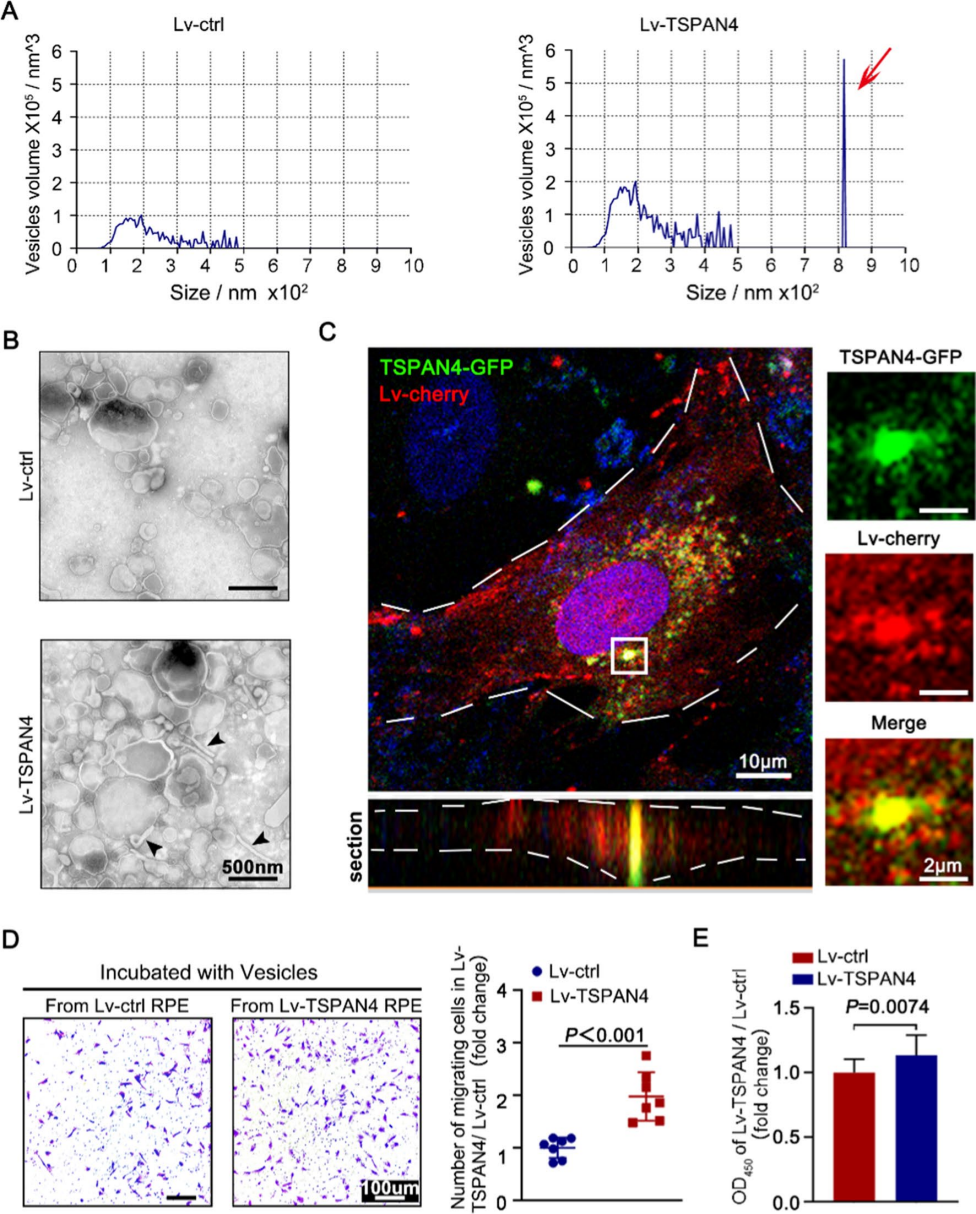
The functional properties of TSPAN4 RPE. RPE were transfected with lentiviral constructs (empty vector as control (Lv-ctrl), or vector overexpressing TSPAN4 (Lv-TSPAN4)). Supernatant of the cultured cells were collected. EVs in the supernatants were isolated by ultracentrifuge. **A** Nanoparticle Tracking Analysis (NTA) of the EVs from Lv-ctrl and Lv-TSPAN4 groups. Vesicles were counted at each 10 diameters. **B** transmission electron microscope (TEM) of collected EVs. Scale bar, 50 μm. **C** Recipient RPE cells were transfected with Lv-mCherry. mCherry^+^ RPE were incubated with collected EVs isolated from TSPAN4-GFP transfected RPE cells and were visualized by confocal microscope. Sectional image confirmed the existence of TSPAN4^+^ migrasomes inside the recipient cell. White line demarcated the mCherry^+^ cell. **D**, **E** RPE were transfected with Lv-ctrl or Lv-TSPAN4. The EVs from these two groups were isolated and added to the culture medium of native RPE. The recipient RPE was trypsinized and subjected to Transwell or CCK8 assay. The migration ability was evaluated by calculating the average number of migrated cells in 5 random vision fields (**D**). Proliferation capability was tested with CCK-8 and measured with OD450 (**E**)

As migrasome is one member of EVs, nanoparticle tracking analysis (NTA) and TEM are utilized to evaluate the characteristics of migrasome. We collected EVs by ultracentrifuging the supernatant of RPE transfected with TSPAN-GFP or vector control. The amount of EVs in each 100 nanometers were shown in Fig. 4A. The result clearly showed the ~ 800 nm EVs are present only in TSPAN4 overexpression group. What’s more, TEM of vesicles showed vesicles attached with tube-like structure, recognized as RFs (black arrowhead), in TSPAN4 overexpression group (Fig. 4B).

Migrasome function of interest was its role in cell–cell communication. Recent research discussed migracytosis in the transportation of RNA [26, 31]. We hypothesize that RPE cells communicate with other RPE cells, at least partially, through migrasomes. Therefore, we collected migrasomes by ultracentrifuge TSPAN-GFP transfected RPE cells and added these migrasome to the supernatant of Lv-mCherry transfected RPE to observe whether RPE cells uptake migrasomes. Images of the mCherry RPE cells showed the uptake of the migrasomes derived from TSPAN4-GFP transfected RPE (Fig. 4C). The diameter of the migrasome was around 1 μm. This result implied that RPE cells may be activated by messages left by migrating RPE cells in PVR. To confirm it, we conducted functional tests of migrasome derived from TSPAN4-overexpressing RPE. Migrasomes from RPE transfected with Lv-TSPAN4 or Lv-ctrl were collected by ultracentrifuge and were added to primary cultured human RPE. Migratory and proliferative capabilities were measured using Transwell and CCK-8 assay, respectively. Results showed that Migrasomes from TSPAN4-overexpressed RPE significantly promote the migration and proliferation of RPE (Fig. 4D, E).

### TSPAN4 contributes to PVR progression in rabbit PVR model

**Fig. 5:**
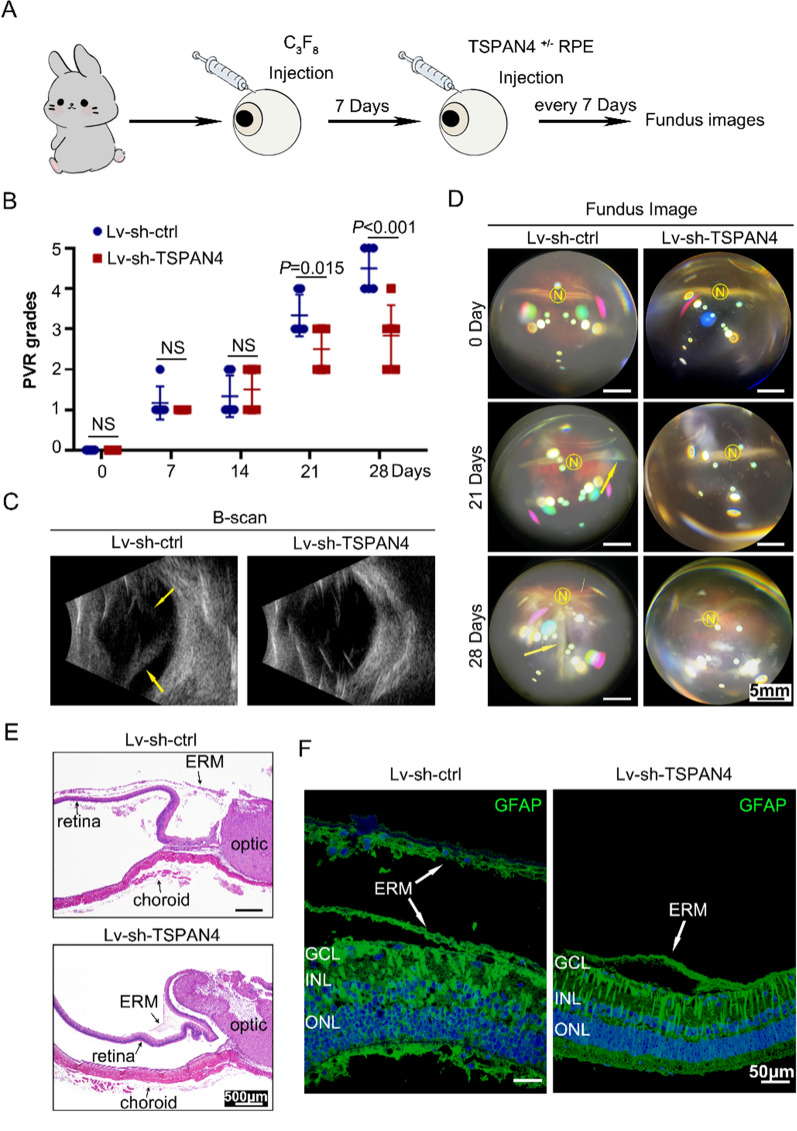
Evaluation of the effect of TSPAN4 on PVR development in a rabbit model. **A** A flow chart of the in vivo rabbit PVR model experiments. Gas vitrectomy was conducted by intravitreal injection of C_3_F_8_. RPEs were transfected with Lv-sh-TSPAN4 to stably silence TSPAN4 or Lv-sh-ctrl as the normal control. Thereafter, these two groups of RPE cells were injected into the vitreous cavity of the rabbits to induce PVR 7 days after gas vitrectomy. **B** PVR grades were evaluated in each group at indicated times based on the fundus image. **C** Representative ultrasound B-scan image at indicated time in both groups. A funnel-shaped retinal detachment can be observed in Lv-sh-ctrl group. The yellow arrows marked the detached retina in the vitreous space. **D** Representative fundus image of rabbits in each group at indicated times. Yellow arrows at wrinkles and folds of the detached retina. **E** HE staining showed detached retina with contractile membranes in Lv-sh-ctrl group, whereas in Lv-sh-TSPAN4 group the ERM is thinner. **F** The immunofluorescence of GFAP in eyeball sections of Lv-sh-ctrl and Lv-sh-TSPAN4 groups indicates the disordered structure of retina and formation of contractile ERM (white arrow) in the control group. N, nerve optic; ONL, outer nuclear layer; INL, inner nuclear layer; GCL, ganglion cell layer; ERM, epiretinal membrane. Values are mean ± s.e.m. NS, is not sufficient

To further confirm the role of migrasome in PVR progression, we established a PVR experimental model using pigmented rabbits. Gas vitrectomy was conducted by intravitreal injection of C_3_F_8_. Lv-TSPAN4-GFP transfected RPE cells were applied in establishing this model. We first transfected these cells with Lv-sh-TSPAN4 to stably silence TSPAN4 or Lv-sh-ctrl as the normal control. Thereafter, these two groups of RPE cells were injected into the vitreous cavity of the rabbits to induce PVR 7 days after gas vitrectomy. Afterward, PVR development was observed every 7 days (Fig. 5 A), and PVR grade was evaluated using the Fastenberg Classification (Additional file 1:  Table S2). The PVR grade of control group is statistically significantly higher after 21 days (*P* < 0.05; Fig. 5B). Representative fundus images and ultrasound B scanning at indicated times were shown in Fig. 5C, D. Results clearly showed in TSPAN4 inhibited group, the severity of the retinal detachment is lessened. In addition, it was discovered that the epiretinal membrane (ERM) in HE-stained eyecup was formed by injected cells and that retina adhesion was compromised by the tractional forces of ERM (Fig. 5E). Finally, considering the retina is a beautifully layered structure where müller cells span the entire thickness of the neuroretina, immunofluorescence of GFAP (the marker of müller cell) was applied to evaluate the regularity of retina. Compared with well-organized retina in Lv-sh-TSPAN4 group, result highlighted the disordered structure in all layers in Lv-sh-ctrl group which indicated a more severe PVR development (Fig. 5F).

### TSPAN4 is induced upon Smad2/3 activation and the bubbling of migrasome is associated with Alix

**Fig. 6:**
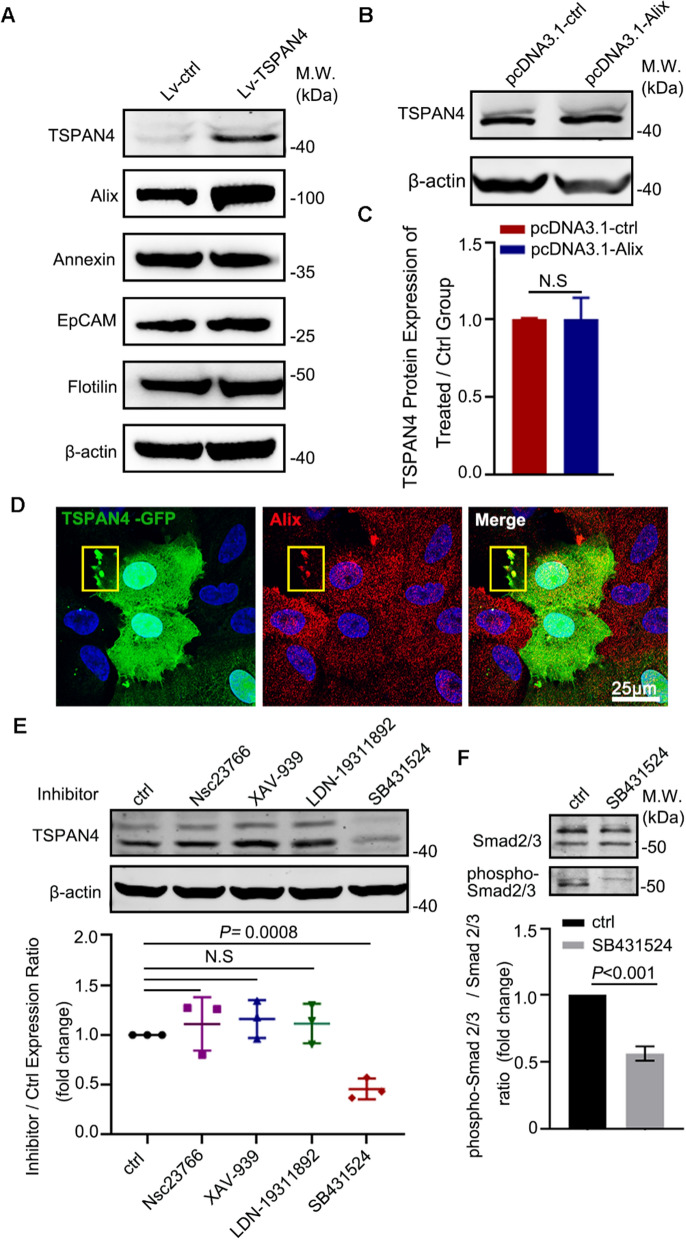
Mechanism of migrasome formation. **A** RPE were transfected with Lv-ctrl or Lv-TSPAN4. Cells were then lysed for western blotting to detect the expression of EVs-secretion related proteins. **B** RPE cells were transfected with Alix overexpression plasmids or control plasmids, and expression of TSPAN4 was detected by western blot. **C** Expression of TSPAN4 detected by western blot was quantified by Image J software. **D** RPE were transfected with Lv-TSPAN4-GFP and were then stained with Alix. Cell were then visualized by a confocal microscope. The white box emphasized the bubbling migrasome. **E** RPE cells were treated with TGF-β1 and LDN-19,311,892 (antagonist of Smad1/5/8), nsc23766 (antagonist of Wnt), XAV-939 (antagonist of Rac), SB431524 (antagonist of Smad2/3). TSPAN4 expression was measured by western blot and quantified below. **F** RPE cells were co-treated with SB431524 and TGF-β1. Smad2/3 and phospho-Smad2/3 were detected by western blot. The Smad2/3 phosphorylation was normalized to levels of total Smad2/3. Values represent Mean ± SEM. N.S, not sufficient

Endosomal sorting complex required for transport (ESCRT) machinery and tetraspanins are molecules known to drive multivesicular body biogenesis by inducing the formation of intraluminal vesicles thereby are often used as markers of EVs, including Alix, Epicam, Annexin, Flotilin, etc. [32–35]. To preliminarily explore the relationship between these proteins and TSPAN4, we examined the expression of these proteins after modulating TSPAN4 expression. Results indicated that overexpressing TSPAN4 in RPE cells upregulated Alix expression (Fig. 6A). Alix was indispensable for receptor sorting into intraluminal vesicles that assist in the sorting and delivery of tetraspanins to exosomes for further release [22, 36]. However, whether Alix is related to migrasome bubbling has not been studied. Alix usually considered only as a pathway to transport EVs and expression rarely being disturbed. Specifically, our western blotting indicated that regulating the expression of TSPAN4 significantly induced the concordant expression of Alix (Additional file 1:  Fig. S2) whereas Alix overexpression did not affect TSPAN4 expression. Therefore, our result indicated TSPAN4 modulates the expression of Alix in RPE cells unidirectionally. The immunofluorescence of TPSAN4 and Alix showed their colocalization close to the bubbling membranes indicating Alix assisted with TSPAN4 for migrasome formation (Fig. 6D).

Thereafter, we tried to explore the mechanism of TGF-β1 induced upregulation of TSPAN4. TGF-β1 activates a variety of powerful signaling pathways (e.g., Smad2/3, Smad1/5/8, Rac, and the Wnt/β-catenin pathways). In TGF-β1 treated RPE, we blocked these key pathways with specific antagonists and observed which pathway regulate the expression of TSPAN4. It was found that treatment with Smad2/3 inhibitors suppressed Smad2/3 phosphorylation and strongly decreased TSPAN4 expression (*P* < 0.001; Fig. 6E, F). This result suggested that TSPAN4 is induced upon phosphorylation of Smad2/3, the most canonical signaling pathway of TGF-β.

## Discussion

**Fig. 7:**
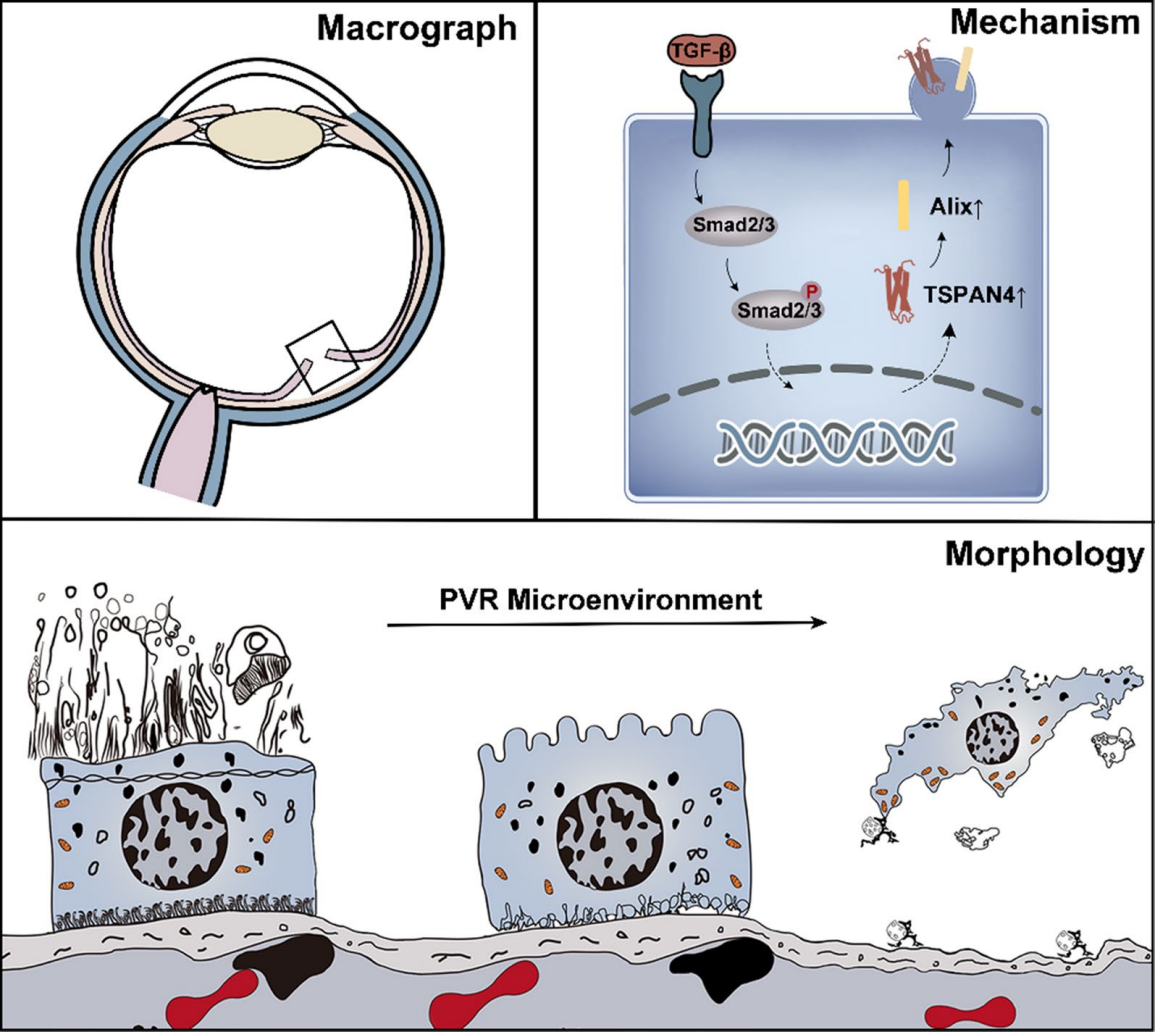
Illustration of migrasome characteristics in proliferative vitreoretinopathy (PVR). Mechanically, the induction of TGF-β1 causes the up-regulation of TSPAN4. The migrasome was then generated in a Alix-associated pathway. Morphologically, the transformation of RPE cells and the formation of vesicles are shown under PVR microenvironment

Our previous understanding of migrasomes is that they are newly discovered vesicular organelles, which exist in migrating cells. By zebrafish embryonic development model, Yu et al. first discovered migrasomes and found migrasomes are important in intercellular communication which conveys spatial and temporal information [23]. Functionally, migrasome guided migrating cells to destined position for organ biogenesis [27, 28]. Nevertheless, whether migrasome contribute to migration process during pathological condition is unknown. Here our study characterized RPE-derived migrasome under PVR microenvironment and confirmed its role in RPE migration and PVR development (Fig. 7). Results in PVR experimental rabbit model found that the inhibition of TSPAN4 expression in RPE significantly abrogated its ability in initiating PVR. Our result is consistent with Yu’s study, which suggested that migrasomes provided regional cues for recipient migrating cells and result to zebrafish organ morphogenesis [27].

As a special type of organelles [23, 37], migrasome is also a subtype of EVs up to 3000 nm in size and which mediate cell migration. TEM described migrasome as the pomegranate-like structure that retraction fibers are pulled out from the trailing edge of a migrating cell [23]. And TSPAN4 was elected as the marker to monitor the migrasome biogenesis process [24]. Accordingly, we used those methods to observe migrasome in RPE cells. TSPAN4 expression have never been studied in retina. Our result portrayed müller cells express TSPAN4 rather than RPE cells in natural state. In PVR membrane clinical samples, we discovered the overexpression of TSPAN4 in RPE cells and the existence of migrasome-like EVs.

Previous studies have suggested that the loss of polarity morphology disappearance is important for EMT progression and is relevant to the remodeling of the RPE microvilli and basal infoldings [3]. Ex vivo models have the superiority of being able to mimic the polarized RPE natural state with the microvilli and basal infoldings. Here our ultrastructural images showed that polarity organelles assist in multi-vesicle formation when RPE cells are activated by TGF-β1. Additionally, most EV studies are performed in vitro and harvested the EVs from cultured cells and subjected to functional studies [38]. These studies may not reflect the spatiotemporal EVs production progress in situ. In contrast, our study presented the spatiotemporal properties of migrasomes production in situ. That is, under EMT, accompanied with RPE migration, RPE lost microvilli and basal infoldings to form multivesicular bodies, which finally evolve into migrasomes. Based on their appearance and size, after comparing our results with previous researches [24, 27], these EVs are recognized as migrasomes.

In normal rat kidney (NRK) epithelial cell lines, overexpression of TSPAN4 enhanced migrasome formation [24]. Accordingly, in our in vitro studies, RFs extension, migrasome formation and degeneration were gradually observed in cells transfected with GFP labeled TSPAN4. These results further confirmed the biogenesis of migrasome is accompanied with the migratory process of RPE. Thereafter, we elaborated on characterizing the progress and mechanism of migrasome bubbling from RPE cells under PVR microenvironment and explored its function. By modulating the expression of TSPAN4, both in vitro and in vivo functionals studies confirmed that migrasome contribute to the activation of RPE and progression of PVR.

The mechanisms of EVs in cell motility is complicated. Small EVs (sEVs), which was previously named exosomes, is a kind of EVs with size of 30**–**150 nm [39, 40]. Compared with migrasomes, our understanding of the formation and function of sEVs is relatively deeper. Exosomes are canonically formed by the inward budding of the early endosomal membrane to form intraluminal vesicles within endosomes. sEVs can carry many types of functional molecules which exert key functions in recipient cells. Our previous research focused on the role of RPE-derived sEVs in activating RPE and promote PVR: RPE cells secreted miR-543-rich exosomes and promote the migration and proliferation of recipient RPE, which contribute to the progression of PVR [41]. Compared with sEVs, much less is known about the biogenesis and function of RPE-derived migrasome. However, inspired by our preceding work, here we modulate the migrasome biogenesis in RPE cell by overexpressing TSPAN4 or stimulating RPE with TGF-β1. Migrasomes were generated at the tips or intersections of retraction fibers at the back of migrating RPE cells. Those vesicles can be collected and ingested into recipient RPE cells and promote its migration. This paracrine-like communication manner in migrasome is similar with that in sEVs. Nevertheless, the biogenesis of migrasome is different with sEVs. Although migrasome, like exosome and other EVs, has been shown to be rich of tetraspanins expression, the exact tetraspanin protein in each EVs is relatively specific. For example, the tetraspanin CD63 is uniquely enriched in sEVs, whereas other tetraspanins (like TSPAN4) control the biogenesis of migrasomes exclusively [42]. Besides, sEVs are derived from ubiquitinated endocytosed cargoes through the ESCRT machinery which results in tetraspanin-enriched lipid microdomains. Migrasome, as large EVs, is considered to be generated by only a partial of ESCRT proteins. Of these, Alix-syndecan-syntenin complex are known to drive its formation [39]. Our immunofluorescence result discovered Alix is co-localized with migrasome in RPE cells. Upregulation of TSPAN4 would increase Alix expression, while upregulation of Alix didn’t alter TSPAN4 expression. These results implied that Alix act as a tool for migrasome formation. With the signal of demand for migrasome formation, Alix is upregulated. While simply up-regulate Alix, this “tool”, does not affect the demand for migrasome formation. To sum up, Alix is involved in migrasome biogenesis when RPE cells treated with TGF-β.

TGF-β activates several intracellular signaling pathways, mainly canonical Smad2/3 signaling, to regulate a diversity of cellular functions. First of all, TGF-β specifically binds to TGF-βRII which then recruits and phosphorylates TGFβRI, leading to the activation of TGF-βRI [43]. Next, activated TGF-βRI recruits and phosphorylates Smad2/3 proteins. Activated Smad2/Smad3 form complexes with the cytosolic Smad4 and these complexes are then translocated to the nucleus to regulate target gene expression. The target genes are mainly associated with cell proliferation, migration, differentiation and death [44, 45]. Conversely, negatively regulating Smad2/3 activity would inhibit these processes. For example, in RPE, inhibiting Smad2/3 signaling hampered the migration and proliferation of RPE and alleviated PVR progression [46]. Inhibited phospho-Smad2/3 by overexpressing Smad7 suppressed migration, proliferation and fibrogenic responses in RPE after retinal detachment in a mice model. What’s more, a Korean research group has found that pirfenidone, a small compound which blocks the nuclear translocation of Smad2/3, inhibits TGF-β1-induced fibrosis process in the human RPE cell line ARPE-19 [47]. Besides, Smad2/3 also regulate other key pathways, such as Wnt [44,48] (a key pathway functioned in maintaining the cytoskeletal arrangement and cell polarity homeostasis), in a context-dependent way. Smad2/3 regulates Wnt pathway by binding TCF and LEF. Blocking Smad2/3 therefore not only elicits cellular responses like migration and proliferation, but may also affect cell polarity through Wnt.

It is well established that TSPAN4-positive migrasome plays important role in PVR progression. In PVR microenvironment, RPE cells were exposed to TGF-β. Afterwards, Smad2/3 signaling pathway is activated and trigger TSPAN4 expression and migrasome formation. TSPAN4 positive migrasome promote the migration and proliferation of RPE cells which finally accelerate PVR membrane formation. Early intervention of RPE activation in high-risk patients is crucial for obtaining better outcomes because once PVR develops, the prognosis is unsatisfactory. Targeting migrasome can be considered as one of an effective way in preventing or treating PVR.

## Conclusion

Here we characterized migrasomes of RPE in PVR microenvironment and revealed its pivotal role in RPE activation and PVR progression. Thus, targeting TSPAN4 or blocking migrasome formation might be a new therapeutic method against PVR.

## Methods

### Reagents and antibodies

The following antibodies were applied: Tetraspanin-4 (NBP1-59438, Novus); RPE 65 (MA1-16578; Thermo Fisher Scientific); GFAP (ab279290, Abcam); CD11b (ab8878, Abcam); integrin α5 (ab288767, Abcam); EOGT (ab190693, Abcam); NDST1 (26203-1-AP, Proteintech); Alix (2171, Cell Signaling); Alix (92,880, Cell Signaling); Smad2/3 (3102, Cell Signaling); Phospho-Smad2/Smad3 (8828, Cell Signaling); Alpha-Smooth Muscle Actin (MA5-11547, Thermo Fisher Scientific); GAPDH (5174, Cell Signaling) and β-actin Rabbit antibodies (ab8227, Abcam). Secondary antibodies used included the following: Alexa Fluor 488 Anti-Mouse; Alexa Fluor 555 Anti-Rabbit; Alexa Fluor 555 Anti-Mouse; and Alexa Fluor 555 Anti-Rabbit (Thermo Fisher Scientific). Additionally, CCK8 (ab228551, Abcam), Dulbecco’s modified Eagle’s medium (DMEM)/F12 culture media, and fetal bovine serum were obtained from Thermo Fisher Scientific.

### RBCCs Preparation and Cell Culture

All animal experiments were performed according to a protocol approved by the Institutional Animal Care and Use Committee (IACUC). Twenty male brown Norway rats (10 weeks old) were purchased from Charles River Laboratory Animal Co. Ltd. (Beijing, China). The rats were sacrificed after one week of feed adaptation. Afterwards, the eyes were harvested. Donated human RBCCs, vitreous humor, and RPE cells were provided by Shanghai Red Cross Eye Bank, and their use conformed to the Declaration of Helsinki. PVR membrane, macular epiretinal membrane, and vitreous samples were provided from surgeries with patient permission (list in Additional file 1:  Table S2).

The RPE cells were isolated from the donated human eyes as described in previous studies [49] To maintain the polarized RPE characteristics, they were used within four passages, each of which were cultured in Dulbecco’s modified Eagle’s medium (DMEM)/F12 culture media with 10% fetal bovine serum (FBS) at 37 °C with 5% CO_2_. To prepare the EMT model, the cells were starved for 12 h, then stimulated with 10 ng/mL TGF-β1(240-B-010/CF, R&D System) for various time intervals.

### Transmission and scanning Electron Microscopy

The rat eyeballs were pierced with a syringe through the corneoscleral limbus. Subsequently, the anterior segment of the eye and neural retina were discarded using a microscope. The RBCCs were washed three times in cold PBS, then incubated in 3D-printed molds placed in 12-well plates. The culture buffers were prepared as follows: Dulbecco’s modified Eagle’s medium (DMEM)/F12 culture media was supplemented with 100 × penicillin, 10% FBS, and 200 ng/ml TGF-β1. Each rat eyecup was incubated with 200 µl of this culture media. The RBCCs plate was stored at 37 °C in a humidified incubator with 5% CO_2_ for specified time intervals. The samples were collected after washing in PBS three times. The RBCCs were placed in an electron microscope fixing solution and fixed at 4 °C overnight for subsequent TEM and SEM testing. The RBCCs were fixed with 4% paraformaldehyde at 4 °C overnight for immunofluorescence.

### Transfection

For stable TSPAN4 overexpression or knockdown, lentivirus-expressing TSPAN4 (Lv-TSPAN4-GFP, Lv-ctrl as control) or TSPAN4-specific shRNAs (Lv-sh-TSPAN4; Lv-sh-ctrl) synthesized by GeneChem (Shanghai, China) were used. The RPE cells were infected at a MOI of 30 according to the manufacturer’s protocol. The sequence of shRNAs in Additional file 1:  Table S3. To transiently inhibit TSPAN4 expression, above specific shRNAs against TSPAN4 were inserted into GV101 vector (sh-Scramble, sh-TSPAN4-1, sh-TSPAN4-2), which was synthesized by GeneChem. Besides, to transiently upregulate Alix expression, shRNAs were inserted into GV417 vector also synthesized by GeneChem. The vectors were transfected with Lipofectamine 3000 (Invitrogen, USA) as the manufacturer’s protocol. The RPE cells were collected 48 h after transfection for further experiments. Protein expression was confirmed by western blotting.

### Real-time quantitative polymerase chain reaction

Total RNA was extracted from cells using TRIzol (Invitrogen, USA) for 5 min at RT, then assessed in the Nanodrop 2000 (Thermo). The RNA was reverse-transcribed using a PrimeScript RT reagent Kit (Takara Clontech). Real-time quantitative PCR was performed in triplicate using a TB Green Premix Ex Taq II kit (Takara Clontech) with a CFX Connect Real-Time System (Bio-Rad) according to the manufacturer’s instructions. RNA expression was normalized to the level of GAPDH. Gene primers were synthesized by Generay Corp., and their sequences are listed in Additional file 1:  Table S4.

### Western blot analysis

RPE cells were washed three times with PBS, then harvested and lysed with a RIPA buffer on ice for 30 min and heated at 100 °C for 10 min. Equivalent amounts of each sample were run on Bis-Tris gels (Epizyme, China). Afterwards, the samples were transferred to nitrocellulose filter membranes, which were blocked in a solution of 5% (wt/vol) skimmed milk powder in PBS for 1 h at room temperature, then probed with a primary antibody at 4 °C overnight. Next, the cells were washed five times in TBST, then incubated with a secondary antibody for 1 h at room temperature. The membranes were imaged using an Odyssey infrared imaging system (LI-COR, USA). The protein expression was quantified with ImageJ (V1.8.0) software. The relative expression was normalized according to the level of β-actin and GAPDH.

### Immunofluorescence analysis

The depigmentation of the RBCCs and the PVR membranes was performed as per the following method. First, the samples were soaked in 1% potassium permanganate for 5 min, then washed three times with PBS, and infused with 2% oxalic acid for 1 min. Next, the samples were washed another three times with PBS. The above procedure was repeated if any pigment remained.

For immunofluorescence, the samples were blocked with 2% BSA for 1 h at room temperature, then incubated with primary antibodies (1:100) at 4 °C overnight. After three rinses with PBS, the samples were incubated for 1 h at room temperature with an Alexa Fluor 488-conjugated or Alexa Fluor 555-conjugated secondary antibody diluted to 1:500 in PBS supplemented with 1% BSA. Afterward, any excess water was removed, and the samples were mounted with Fluoroshield (Abcam, 104,139) on a microscope slide to ensure that the RPE cells faced the microscope and were visualized under a confocal microscope (A1, Nikon, Japan). Uncompressed images were minimally processed with ImageJ software (ver 1.52v; National Institutes of Health).

### Time-lapse imaging

RPE cells were cultured in 35 mm live-cell dishes coated with fibronectin (10 µM/ml). At the density up to 60%, those cells were transfected with Lv-TSPAN4-GFP and time-lapse images were acquired after 24 h by NIKON A1 confocal microscopes.

### EVs Collection

RPE cells transfected with TSPAN4-GFP lentivirus were cultured, and the supernatant was collected. The collected supernatant was centrifuged at 1000 ×*g* for 10 min to discard cells and centrifuged at 28,000 ×*g* for 70 min to collect vesicles. Those vesicles were lysed with RIPA for western blots. Besides, they were also resuspended on PBS for NTA and TEM. NTA were applied by Ribobio (Guangzhou, China). In addition, vesicles resuspended on DMEM for further experiment.

### RPE Migration and Proliferation assays

For migration assay, cells at the density of 1 × 10^5^ cells/ml were seeded in the upper chamber of 24-well Transwell plates (8 μm pore size; Costar, Conning, CA, USA) coating with collected EVs (Lv- TSPAN4/ Lv-ctrl) and 10% FBS in DMEM. The lower chamber was filled with 10% FBS DMEM and incubated at 37 °C for 24 h. Afterwards, the cells were fixed with PFA, and the dorsal membrane was stained with 0.1% crystal violet for 30 min. The stained area was counted in 5 random fields and measured with ImageJ software (ver 1.52v).

For the proliferation assays, cells at a density of 1 × 10^4^ cells/well were seeded in 96-well plates and incubated at 37 ℃ with EVs (Lv- TSPAN4/ Lv-ctrl). After 24 h, the cells were tested with Cell Counting Kit 8 (WST-8 / CCK8; ab228554, Abcam, USA), and the absorbance was measured at 460 nm using a plate reader (Thermo, USA).

### Phagocytosis test

Above collected EVs were resuspended with DMEM/F12 containing 1% FBS and incubated RPE cells were transfected with Lv-mCherry for 24 h. Afterwards, cells were fixed and stained with DAPI. Images were acquired with a NIKON A1 confocal microscope.

### Rabbit models

Eight pigmented rabbits (2 kg, male) were housed at the Animal Center of Shanghai Tenth People’s Hospital. The rabbit experiments were conducted in compliance with IACUC. Preparation of rabbit PVR model followed that of a previous study [50]. All experiments were conducted in the right eye. On the first day, 0.40 ml perfluoropropane (C3F8) gas was injected into the vitreous body using a 30-gauge needle, 2.5 mm from the limbus. After 7 days, the transfected RPE (passage 3) cells and 150 ng PDGF-BB suspended in 0.1 ml PBS (concentration of 30,0000 cells/100 µl) were injected intravitreously using a 27-gauge needle at the rabbit eyes. The control eyes were injected with 0.1 ml PBS without RPE cells. Ophthalmoscopic examinations, including ultrasound B scanning, were applied, and fundus photographs were recorded on days 0, 7, 14, 21, and 28. The degree of PVR was graded using Fastenberg’s classification (Additional file 1:  Table S1). The rabbits were sacrificed on the day 28. Afterwards, the eyes were collected and fixed in a fixation buffer (Servicebio, Shanghai, China). Next, paraffin embedment and serial Sections. (10 μm) were performed, respectively. Observation of the rabbit retinal morphology occurred with the assistance of the HE staining. Immunofluorescence using GFAP was used to evaluate the physiology of retina and the degree of ERM.

### Inhibitors in signaling

Inhibitors were purchased from Selleckchem (TX, USA). Following inhibitors were applied: LDN-19,311,892(0.5 µmol/ml, targeting Smad1/5/8), nsc23766 (50 µmol/ml, targeting Wnt pathway), SB431524 (10 µmol/ml, targeting Smad2/3 pathway), XAV-939 (20 µmol/ml, targeting Rac1 pathway). RPE cells seeded in 6-well plates at a density of 60%. RPE were starved for 12 h, followed by incubation with different inhibitors along with 10 ng/ml TGF-β1 for 24 h. Protein was extracted for western blotting.

### Statistical analysis

All experiments were performed at least three times. The mean and standard error of the mean were calculated for all measured parameters. A value of *P* < 0.05 was considered statistically significant. The data were analyzed using one-way ANOVA, with a Bonferroni correction for multiple comparisons statistical software program SPSS 20.0 (Chicago, IL). Graphs were made with GraphPad Prism 6 software.

## Supplementary Information

**Additional fila 1: Fig. S1.** Detection of migrasome-specific markers from cell bodies and supernatant from RPE cells. (A) RPE cells were transfected with lentiviral constructs (empty vector as control (Lv-ctrl), or vector overexpressing TSPAN4 (Lv-TSPAN4)). The supernatant of the cultured cells was collected. EVs in the supernatants were isolated by ultracentrifuge and lysed by RIPA. The protein expression was analyzed by western blots using the migrasome-specific antibodies. (B) RPE cells that overexpressed TSPAN4 were transfected with sh-TSPAN4 to knockdown TSPAN4 and analyzed by western blot. Sh-Scb as the control group. The expression of related proteins detected by western blot was quantified by Image J software. **Fig. S2.** Detection of Alix expression from overexpressed and downregulated TSPAN4 in RPE cells. (A) RPE cells were overexpressed TSPAN4 (Lv-TSPAN4) by lentivirus. Lv-ctrl as the control group. RPE cells were downregulated TSPAN4(sh-TSPAN4-1, sh-TSPAN4-2) by plasmids. sh-Scb as the control group. Samples were analyzed by western blotting using Alix. (B) Quantification of western blot. **Table S1.** Fastenberg classification for the stage of PVR in rabbit eye. **Table S2.**Clinical samples provided by donar and surgery patients. **Table S3.** Sequence of shRNA. **Table S4.**Primer of target gene.

## Data Availability

Data sharing availability to this article.

## Abbreviations

DFs: Dorsal forerunner cells
EMT: Epithelial-mesenchymal transition
ESCRT: Endosomal sorting complex required for transport
EVs: Extracellular vesicles
IERM: Idiopathic macular epiretinal membrane
NTA: Nanoparticle tracking analysis
PVR: Proliferative vitreoretinopathy
RBCC: RPE–Bruch’s membrane–choriocapillaris complex
RFs: Retraction fibers
RPE: Retinal pigmented epithelium
RRD: Rhegmatogenous retinal detachment
SEM: Scanning electron microscope
TEM: Transmission electron microscope
TSPAN4: Tetraspanin-4
