# Supplementary material for: TSPAN4-positive migrasome derived from retinal pigmented epithelium cells contributes to the development of proliferative vitreoretinopathy
Source: J Nanobiotechnology. 2022 Dec 9;20:519. doi: 10.1186/s12951-022-01732-y (PMC9733225; doi:10.1186/s12951-022-01732-y)

**Supplementary Figure 1**


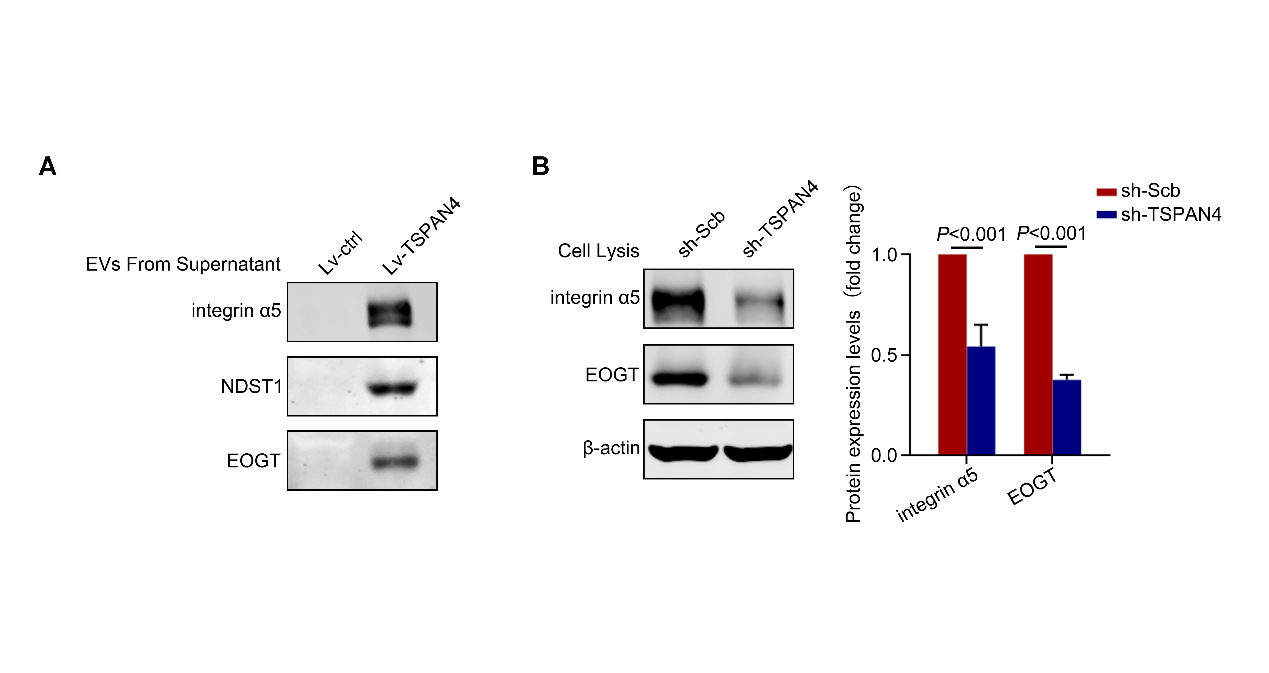
**Supplementary Figure 1. Detection of migrasome-specific markers from cell bodies and supernatant from RPE cells.** (A) RPE cells were transfected with lentiviral constructs (empty vector as control (Lv-ctrl), or vector overexpressing TSPAN4 (Lv-TSPAN4)). The supernatant of the cultured cells was collected. EVs in the supernatants were isolated by ultracentrifuge and lysed by RIPA. The protein expression was analyzed by western blots using the migrasome-specific antibodies. (B) RPE cells that overexpressed TSPAN4 were transfected with sh-TSPAN4 to knockdown TSPAN4 and analyzed by western blot. Sh-Scb as the control group. The expression of related proteins detected by western blot was quantified by Image J software.

**Supplementary Figure 2**


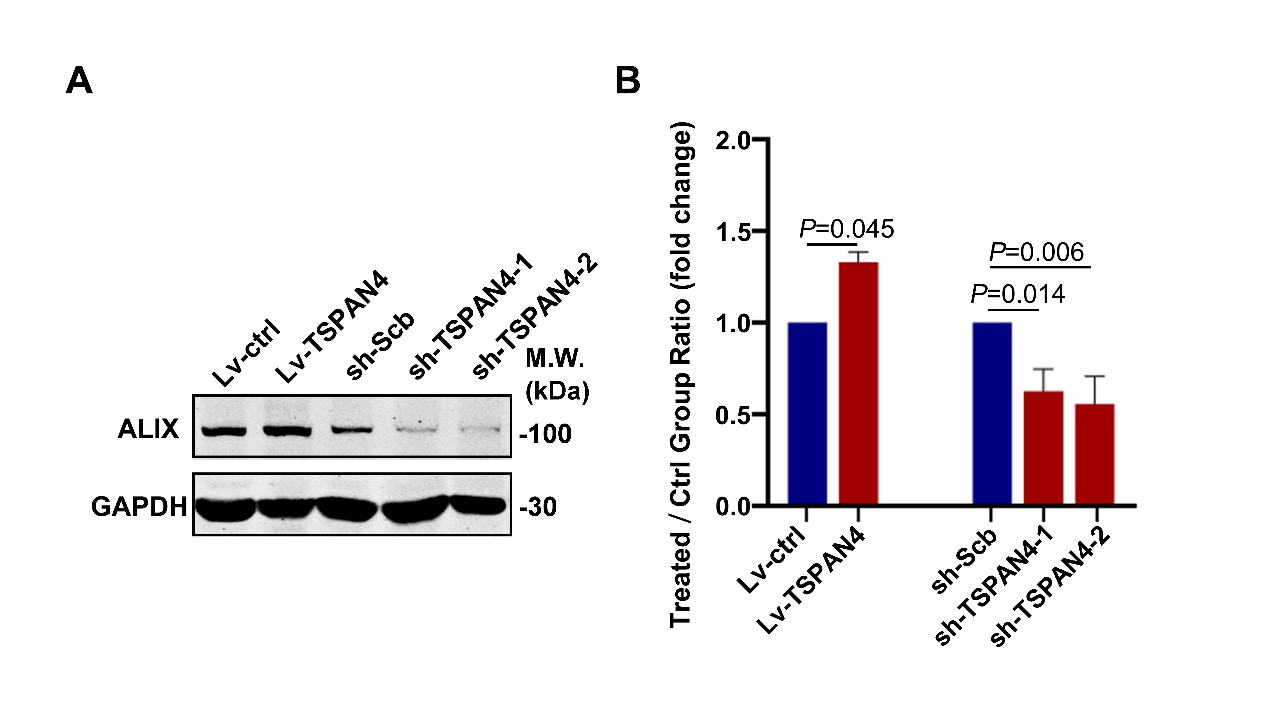


**Supplementary Figure 2. Detection of Alix expression from overexpressed and downregulated TSPAN4 in RPE cells.** (A) RPE cells were overexpressed TSPAN4 (Lv-TSPAN4) by lentivirus. Lv-ctrl as the control group. RPE cells were downregulated TSPAN4(sh-TSPAN4-1, sh-TSPAN4-2) by plasmids. sh-Scb as the control group. Samples were analyzed by western blotting using Alix. (B) Quantification of western blot.


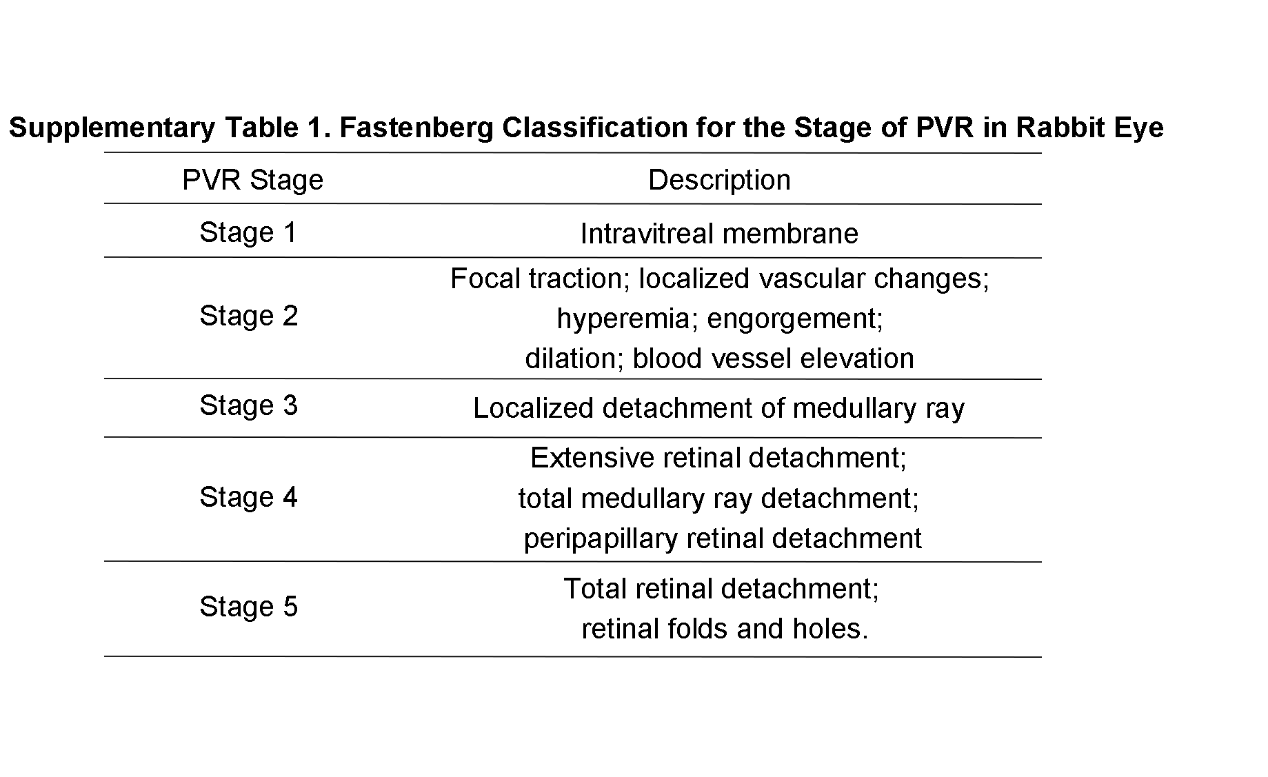

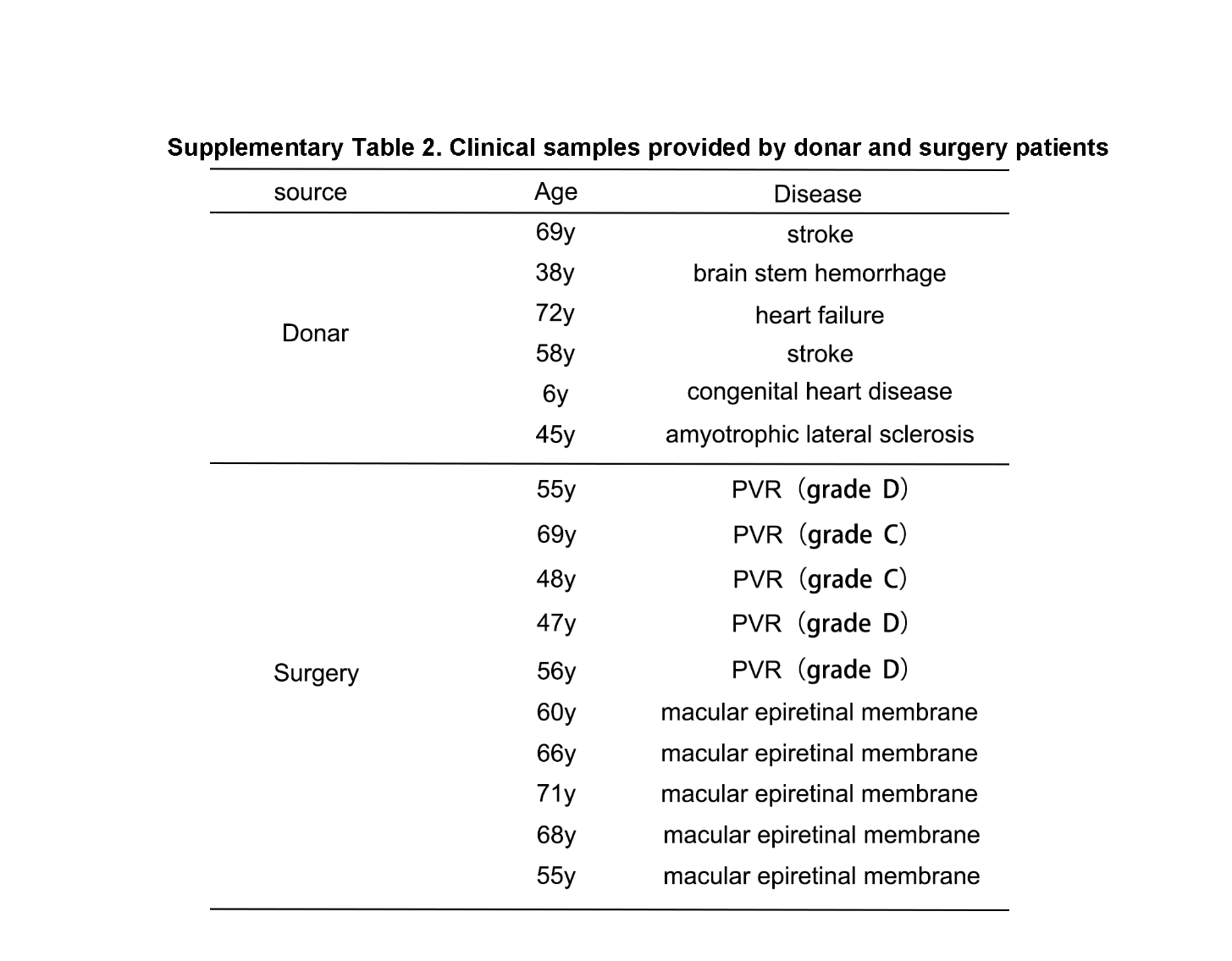

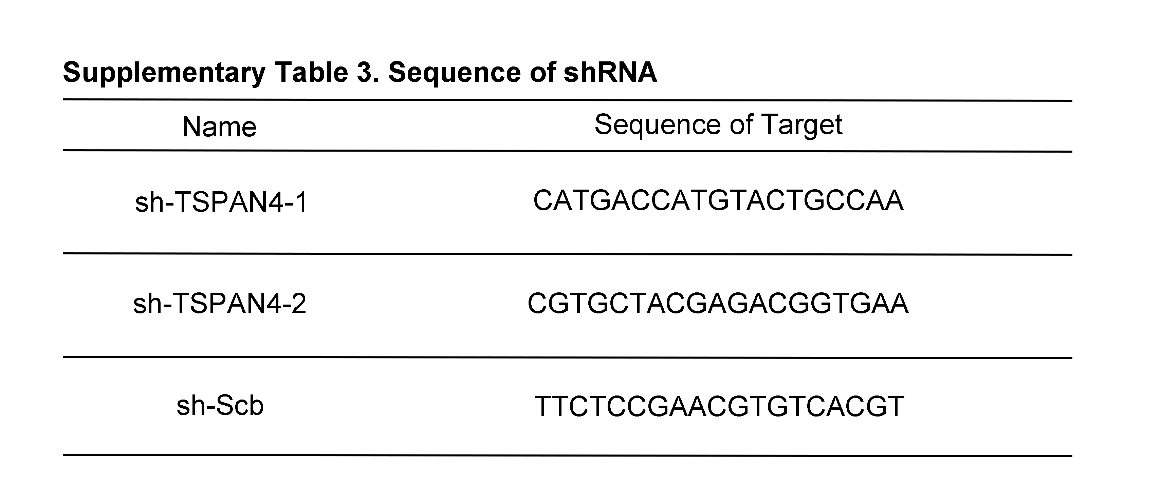

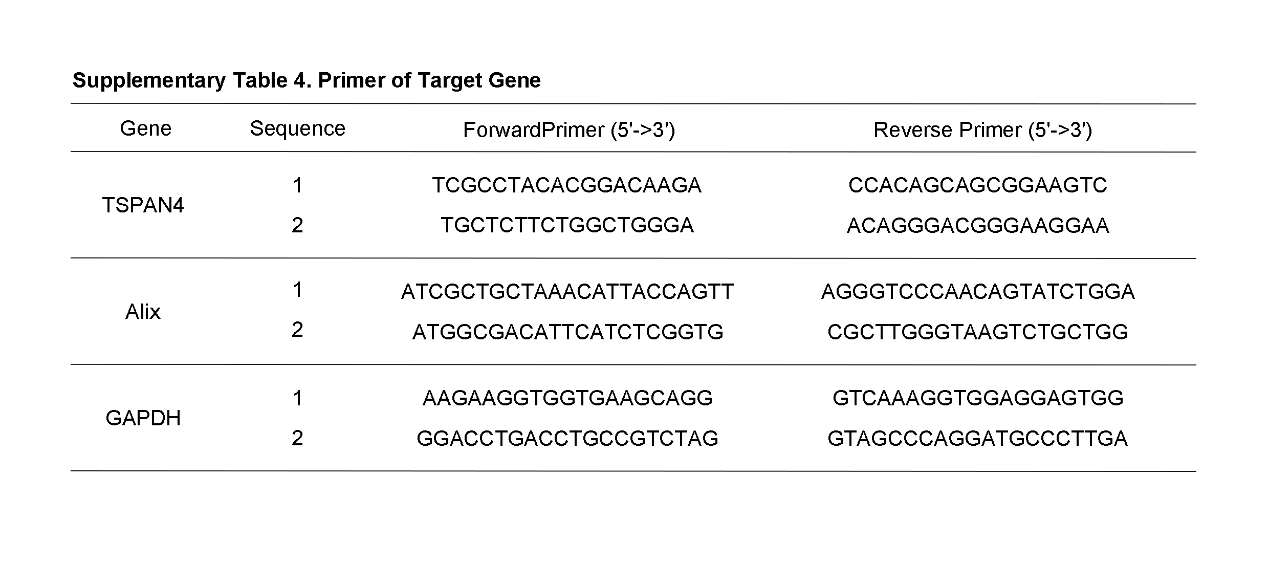

Supplement: Supplementary file 1 — Additional fila 1: Fig. S1. Detection of migrasome-specific markers from cell bodies and supernatant from RPE cells. (A) RPE cells were transfected with lentiviral constructs (empty vector as control (Lv-ctrl), or vector overexpressing TSPAN4 (Lv-TSPAN4)). The supernatant of the cultured cells was collected. EVs in the supernatants were isolated by ultracentrifuge and lysed by RIPA. The protein expression was analyzed by western blots using the migrasome-specific antibodies. (B) RPE cells that overexpressed TSPAN4 were transfected with sh-TSPAN4 to knockdown TSPAN4 and analyzed by western blot. Sh-Scb as the control group. The expression of related proteins detected by western blot was quantified by Image J software. Fig. S2. Detection of Alix expression from overexpressed and downregulated TSPAN4 in RPE cells. (A) RPE cells were overexpressed TSPAN4 (Lv-TSPAN4) by lentivirus. Lv-ctrl as the control group. RPE cells were downregulated TSPAN4(sh-TSPAN4-1, sh-TSPAN4-2) by plasmids. sh-Scb as the control group. Samples were analyzed by western blotting using Alix. (B) Quantification of western blot. Table S1. Fastenberg classification for the stage of PVR in rabbit eye. Table S2.Clinical samples provided by donar and surgery patients. Table S3. Sequence of shRNA. Table S4.Primer of target gene. [file 12951_2022_1732_MOESM1_ESM.docx]
